# Depression and Anxiety Changes Associated with Matched Increases in Physical Activity in Education-, Self-Regulation-, and Self-Regulation Plus Relaxation-Based Obesity Treatments in Women: A Pilot Study Investigating Implications for Controlling Emotional Eating

**DOI:** 10.3390/nu17152475

**Published:** 2025-07-29

**Authors:** James J. Annesi, Steven B. Machek

**Affiliations:** 1 Kinesiology Department, California State University, Monterey Bay, Seaside, CA 93955, USA; smachek@csumb.edu; 2 Mind Body Wellbeing, LLC, Manahawkin, NJ 08050, USA

**Keywords:** emotional eating, physical activity, depression, anxiety, self-regulation, self-efficacy, obesity, treatment

## Abstract

**Background/Objectives:** Improvements in depression and anxiety, associated with moderate increases in physical activity, might induce reductions in emotional eating, especially in women with obesity, where emotion-driven eating is highly problematic. This pilot, field-based study sought to assess whether physical activity increase, itself, primarily predicts improved mood (biochemical theories) or if psychosocial factors associated with cognitive–behavioral treatment are principal correlates (behavioral theories). An aim was to inform improved treatment processes. **Methods:** Women with obesity participated in 6-month community-based behavioral obesity treatments emphasizing either: (a) standard education in weight-reduction methods (*n* = 28), (b) eating-related self-regulation methods (*n* = 24), or (c) self-regulation + relaxation training (*n* = 24). They completed a series of behavioral and psychological self-reports at baseline and Months 3 and 6. **Results:** Findings confirmed no significant difference in 3-month increases in physical activity, by group. There were significantly greater overall improvements in depression, emotional eating, self-regulation, and self-efficacy across the two self-regulation-focused groups (*ps* < 0.02), with anxiety improvement not reaching significance (*p* = 0.055). Separate significant paths from 3-month changes in depression and anxiety → self-efficacy change → emotional eating change were found. The same significant path was detected emanating from 6-month anxiety change; however, the hypothesized path of 6-month changes in depression → self-regulation → self-efficacy → emotional eating was, rather, significant. Weight reduction was considerably greater in the two self-regulation-based groups (~6% reduction), with simultaneously entered changes in self-regulation and self-efficacy significant predictors of those weight changes. **Conclusions:** Findings suggested viability in behavioral theory-driven explanations of the physical activity-mood improvement relationship. Future treatment foci on self-regulatory skills development leading to improvements in eating-related self-efficacy, emotional eating, and weight were suggested to extend the findings of this pilot study.

## 1. Introduction

In the United States (U.S.), obesity—designated as a body mass index (BMI) ≥ 30 kg/m^2^—continues a decades-long increase to currently affecting 46% of women 25 years-of-age and older. Because this upward trend is projected to persist to approximately 60% by 2050 [1], improved treatment is urgently required; preferably with large-scale applicability. Emotional eating is a major psychosocial predictor of excess body weight [2]. This is especially true for women, where psychological/mental health factors associated with weight and weight management are the most prominent [3]. Research suggests that heightened anxiety and depression are strong correlates of emotional eating [4].

Although some positive effects were found, psychotherapeutic treatments of emotional eating within weight-management contexts have generally demonstrated unconvincing outcomes, small effect sizes, and are time-intensive and expensive [5,6,7]. Also, because of the considerable amount of resources required to access such individualized therapy, only a small minority in need stand to benefit from them. The current trend of treating obesity through lifelong pharmacological means or surgery [8,9], may be a response to a general inability to effectively deal with overeating issues through less-invasive behavior-change methods [10].

Increased physical activity is a common component of behavioral obesity treatments, and is reliably associated with reductions in anxiety, depression, and overall negative mood; with large effect sizes [11,12]. The physical activity amount associated with those mood improvements involves an increase of (the equivalent of) ~3 moderate walks/week [13], with no physical activity dose–mood improvement response relationship after such a moderate rise is attained [14]. This is important because high amounts of physical activity have been associated with discomfort and attrition, especially in adults with obesity [15,16,17]; and accelerometry indicates that <5% of U.S. adults were able to sustain the recommended ≥150 min (i.e., 5–7 moderate bouts) per week [18].

Mood improvements have been related to reductions in emotional eating [19], even within large-scale field settings targeting weight reduction [17]. Such approaches have the potential of economically and efficiently addressing emotional eating. However, there are a number of research gaps related to those relationships that should first be addressed. They include an improved understanding of the biochemical and behavioral bases of both physical activity-associated changes in mood and their relationship with emotional eating and weight loss. This shortcoming is exacerbated because relevant studies have primarily been cross-sectional and, thus, have not accounted well for over-time changes [20].

Physiological/biochemical theories of the physical activity–mood change relationship posit that biochemical changes associated with increased physical activity induce improvements in mood, directly (e.g., endorphin hypothesis, serotonin hypothesis, norepinephrine hypothesis, brain-derived neurotrophic factor hypothesis) [11,21]. Consistent with such propositions, increased physical activity would, itself, promote improved mood aside from intervention foci. Thus, basic information-based treatments, that are presently the most prevalent [10], would be adequate for mood, emotional eating, and weight changes as long as the physical activity increase reaches the aforementioned threshold.

Behavioral theories of the physical activity–mood change relationship suggest, rather, that psychosocial mechanisms such as feelings of ability to self-regulate through behavioral challenges induce improvements in mood [11]. Individuals’ ability to internalize self-regulatory skills to maintain goal-directed behaviors through personal/environmental challenges is consistent with social cognitive theory [22,23] and self-regulation theory [24]. Additionally, self-efficacy theory [25] posits that such success with self-managing through lifestyle barriers leads to increased feelings of competence (i.e., self-efficacy) which is predictive of improved mood and overall mental wellbeing. Thus, treatments that focus on building participants’ self-regulatory skills and self-efficacy would hold additional benefit beyond increased physical activity. Because cognitive–behavioral weight-loss interventions that focus on those internal states have also yielded greater increases in their participants’ physical activity (than educational formats; Blinded), it is unclear whether physical activity, specifically, or its combination with such psychosocial changes, best accounts for improvements in anxiety and depression [20]. Additionally, training participants in mood-adjustment methods might further broaden improvements in anxiety and depression, but direct testing of that is lacking [26].

Thus, to extend the available related research and address recent appeals to better understand: (a) the biochemical and psychosocial implications of the physical activity increase-anxiety and depression reduction relationship; (b) the relationship of a set range of physical activity increases with mood change, while accounting for various behavioral treatment types; (c) the degree theory-driven psychosocial mechanisms (e.g., change in self-regulation leading to self-efficacy change) mediate relationships between changes in mood and emotional eating; and (d) treatment and psychosocial implications for short- and longer-term weight loss [4,27,28,29,30]; the present preliminary, field-based research was carried out.

A community-based health-promotion setting with women with obesity, who increased their physical activity by at least (the equivalent of) three moderate walking sessions/week, incorporated multiple treatment formats to address hypotheses and research questions derived specifically for this research. They are as follows:

**Hypothesis** **1.** *Improvements in depression, anxiety, emotional eating, self-regulation of eating, and self-efficacy for controlling eating will significantly improve, overall; however, whether those improvements will significantly differ by treatment group format was left as a research question.*

**Hypothesis** **2.** *Relationships between changes in depression and anxiety, and emotional eating changes, will be mediated by changes in self-regulation of eating leading to self-efficacy for controlling eating changes.*

Follow-up analyses will contrast lost weight, by treatment group. It was hoped that the results clarify relationships between treatment type, psychosocial changes, and weight changes through a theoretical lens so that obesity treatment contents benefit. It was also hoped that the present field setting enables findings to maximize generalizability for rapid applications.

## 2. Materials and Methods

### 2.1. Participants

Participant data were from ongoing field research on community-based weight-management approaches in the U.S. [17]. Their foci differed from those within the present investigation, which had additional inclusion criteria based on the present research aims. Data were from 2018 to 2020, although the final self-reported weight included 2021 (when the COVID-19 pandemic restricted in-person contact). The volunteer women with obesity (BMI ≥ 30 kg/m^2^) were obtained via their phone responses to advertisements in local print and internet media. In addition to obesity, inclusion prerequisites were no reported medical contraindication for safe and full participation, no change in a psychotropic medication and no involvement in another weight-management process (including self-help) during the previous 6 months, and a baseline—Month 3 increase in physical activity (the equivalent of) ≥3 days/week of normal-paced walking (i.e., a weekly increase of ≥9 metabolic equivalents [METS, a measure of physical exertion, also see the physical activity measure below]). Block randomization of participants to the three treatment conditions was associated with the participating community health facilities. Participants in the educational methods (*n* = 28), self-regulation methods (*n* = 24), and self-regulation + relaxation-based (*n* = 24) conditions did not significantly differ on age, BMI, racial/ethnic make-up, yearly family income, educational level, and number who dropped out prior to study start (*n* = 2 for each group; Table 1). A university institutional review board approved the study protocol and the informed consent process requiring a signature from each participant. Ethical and privacy requirements of the World Medical Association Helsinki Declaration and the American Psychological Association were followed throughout.

### 2.2. Measures

Brief, but adequately validated, scales were administered to minimize response burden and subsequent inaccuracies linked to responding to a lengthy battery of instruments [31].

Physical activity was measured using the Leisure-Time Physical Activity Questionnaire [32]. Completed 15 min or longer bouts of “mild” (e.g., normal-paced walking), “moderate” (e.g., fast walking), and “strenuous” (e.g., running) intensities during the previous 7 days were recalled and each assigned their corresponding value of 3, 5, or 9 METs, respectively. Those scores were then summed. Concurrent validity was indicated through correspondences with accelerometry, treadmill testing, and body composition results; with 2-week test–retest reliability reported at 0.74 [33,34,35,36,37].

Depression and anxiety were measured using the corresponding 5 items, each, of the depression/dejection and anxiety/tension scales of the Profile of Mood States-Brief Form [38]. Items required respondents to reflect on overall feelings (e.g., “sad,” “tense,”, respectively) during the previous 7 days, including the present day. Response options ranged from 0 (*not at all*) to 4 (*extremely*), which were summed. Internal consistencies with women on the depression and anxiety scales were reported as Cronbach’s αs = 0.95 and 0.90, respectively (study sample Cronbach’s αs = 0.87 and 0.89, respectively). The 3-week test–retest reliability was reported at 0.74 and 0.70, respectively [38].

Emotional eating was measured using 15 items of the Emotional Eating Scale [39]. Propensity to eat when prompted by a challenging feeling state (e.g., “sad,” “frustrated”) had response options that ranged from 0 (*no desire to eat*) to 4 (*an overwhelming urge to eat*), which were summed. Internal consistencies were reported to range from Cronbach’s α = 0.72–0.79 (study sample Cronbach’s α = 0.72). The 3-week test–retest reliability was reported at 0.79 [39].

Self-regulation of eating was measured using the 10 items of the Eating-Related Self-Regulation Scale (e.g., “When I get off-track with my eating plans, I work to quickly get back to my routine”) [40]. Response options ranged from 1 (*never*) to 4 (*often*), which were summed. The internal consistency was reported at Cronbach’s α = 0.79 (study sample Cronbach’s α = 0.74), and 2-week test–retest reliability was reported at 0.78 [40].

Self-efficacy for controlling eating was measured using the 20 items of the Weight Efficacy Lifestyle Scale [41]. The respondent’s confidence in their ability to control eating under challenging conditions/stimuli such as social pressure to eat and high food availabilities (e.g., “I can resist eating even when others are pressuring me to eat,” “I can resist eating even when high-calorie foods are available”) had response options that ranged from 0 (*not confident*) to 9 (*very confident*). Those responses were summed. Reported internal consistencies ranged from Cronbach’s α = 0.70–0.90 [41] (study sample Cronbach’s α = 0.74).

Participants’ weight was measured using a digital floor scale (Health-o-Meter Model 80 kl; McCook, IL, USA) calibrated the same day of each assessment. Measurements were recorded to the nearest 0.10 kg. Heavy outer-clothing and shoes were removed by participants prior to measurements. Non-instructional study staff completed measurements through Month 12, while weight at Month 24 was self-reported by each participant due to the COVID-19 restriction of in-person contact.

### 2.3. Procedure

Treatment instructors for this pilot study were staff members of the participating community facilities and trained by study staff in only their randomly assigned treatment. Contents for each treatment type was adapted from National Institutes of Health/National Cancer Institute-certified programs [42]. Each of the three treatment types lasted 6 months and totaled 11 participant/hours during that period.

The educational methods treatment was guided by the health belief model [43]. That paradigm suggests that individuals seeking a goal such as weight loss will improve their control over physical activity and eating through education on their benefits and instructions on carrying out such behaviors. After an initial one-on-one meeting that briefly reviewed the overall program contents, topic-specific written materials were supplied to this group’s participants every 2 weeks, to be reviewed individually. The contents of each set of those materials was adapted from the LEARN Program [44], and supported within 3 days by a one-on-one contact with an educational treatment instructor throughout the 6 treatment months. Instructional topics included the benefits of physical activity, healthy eating, proper exercise methods, and fruit/vegetables consumption [45,46]; and the demonstrated disadvantages of excessive amounts of sweets [47]. Limiting daily energy intake to 1200–1500 kcal/day was indicated as a range for safe weight loss for most adults.

Both the self-regulation treatment and self-regulation + relaxation treatments were guided by Bandura’s social cognitive and self-efficacy theories [22,25], and self-regulation theory [24]. Their key tenets emphasize interrelations between environmental, psychological, and behavioral factors within treatments and also highlight one’s ability to overcome barriers toward behavioral changes via use of self-regulatory skills yielding increased feelings of ability/mastery (i.e., self-efficacy). The self-regulation + relaxation treatment included relaxation-based treatments such as deep breathing and abbreviated progressive muscle relaxation [48] during ~20% of the allocated treatment time. Recent research indicated positive effects of progressive muscle relaxation on both anxiety and depression, as well as possible increased effects when combined with other treatment components [26]. Both the self-regulation treatment and the self-regulation + relaxation treatment initially supported physical activity primarily through one-on-one instruction in self-regulating through barriers such as boredom and discomfort using self-regulatory skills suggested by Michie et al. [49]. These included cognitive restructuring, relapse prevention, and dissociation from discomfort. Also, proximal goal-setting and tracking/acknowledging even minor amounts of progress were emphasized in efforts to increase participants’ self-efficacy. Starting at Month 2, the same array of self-regulatory skills was systematically adapted to also help participants control their eating. Eating behavior changes focused primarily upon increasing fruit/vegetable consumption, reducing sweets, and limiting daily energy intake to 1200–1500 kcal/day, based on current weight. Sessions focused on eating changes were primarily conducted in small groups. Weekly self-weighing was encouraged for both self-regulation groups, but weights were not shared with other participants.

Participants in each of the treatment groups were referred to the website “myplate.gov” for additional nutrition information, as they desired such. Physical activity types were self-selected by each participant. The governmental recommendation of ≥150 min/week of physical activity for health benefits [50] was mentioned to all participants, but it was also suggested that *any* increase could be beneficial. Within each treatment condition, emotional eating was addressed in only a cursory manner, which served to minimize expectation effects on that factor [51]. Treatment fidelity monitoring was completed by non-instructional staff on 10% of sessions using a series of protocol compliance-related items requiring their Likert scale-type responses. Scores indicated strong treatment protocol compliance, requiring only minor adjustments that were provided to instructors by the raters. Non-instructional staff also administered study measures to participants in a private area.

### 2.4. Data Analyses

Based on criteria suggested by White et al. [52], there was no systematic bias in the presence/absence of the overall 14% of missing data, all being beyond baseline. That missing-at-random condition fulfilled a required provision for imputation using the expectation-maximization algorithm [53,54]. Thus, the desired intention-to-treat format was enabled. An overall sample size of 67 was required to detect a moderate effect of Cohen’s *f*^2^ = 0.15 (indicated in previous related research; Blinded) at the power level of 0.80, α < 0.05 [55]. Variance inflation factor scores <2.0 (tolerance values >0.50) indicated satisfactory multicollinearity in the data, with no observed floor or ceiling effects.

To address Hypothesis 1, mixed-model repeated-measures analyses of variance (ANOVAs) assessed gain (change) scores in physical activity, depression, anxiety, emotional eating, self-regulation of eating, and self-efficacy for controlling eating both overall, and on their change × treatment type interactions over 6 months. Follow-up contrasts of baseline—Month 3 and baseline—Month 6 changes, by treatment group, incorporated the Least Significant Difference method (no adjustment for multiple tests).

To enable a sufficient sample size, the mediation path models incorporated data aggregated across groups. To address Hypothesis 2, a series of mediation paths were fit from changes (over both 3 and 6 months) in the mood scores toward emotional eating changes, through changes in self-regulation of eating leading to self-efficacy for controlling eating changes. Finally, a sensitivity analysis contrasted changes in weight, by group, over 3, 6, 12, and 24 months.

For group contrasts, statistical significance was set at α < 0.05 (two-tailed). Because prior research, which included approximately 150 findings in total, indicated directionality in relationships among changes in the tested psychosocial variables [17,56], one-tailed tests were used to assess significance within the regression analyses. Statistical analyses were completed using SPSS Statistics Version 28.0. The PROCESS 4.2 macroinstruction Model 6 for serial mediation incorporated 10,000 percentile-based bootstrapped re-samples of the data [57].

## 3. Results

### 3.1. Score Changes, Across Treatment Types

Descriptive statistics are given in Table 2, by group, including all participant data aggregated (*N* = 76). For aggregated data, Table 1 also includes ANOVAs assessing significance of both overall change, and change × treatment type. Overall, there were statistically significant improvements on all tested psychosocial variables. Significant time × treatment group interactions were found on depression, emotional eating, self-regulation of eating, and self-efficacy for controlling eating. The time × treatment group interaction with anxiety did not reach statistical significance (*p* = 0.055; Table 2). Follow-up contrasts indicated that for baseline–Month 3 changes, emotional eating and self-efficacy for controlling eating improvements were significantly more pronounced in the self-regulation- and self-regulation + relaxation-based treatments than in the educational treatment. For baseline—Month 6 changes, improvements in each of the psychosocial variables were significantly greater in the self-regulation and self-regulation + relaxation treatments than in the educational treatment. Results associated with the two self-regulation conditions did not significantly differ from one another during either temporal period. There were no significant differences in changes in physical activity, as change in that variable was a participant inclusion criterion.

### 3.2. Regression Analyses Predicting Emotional Eating Changes

**Figure 1:**
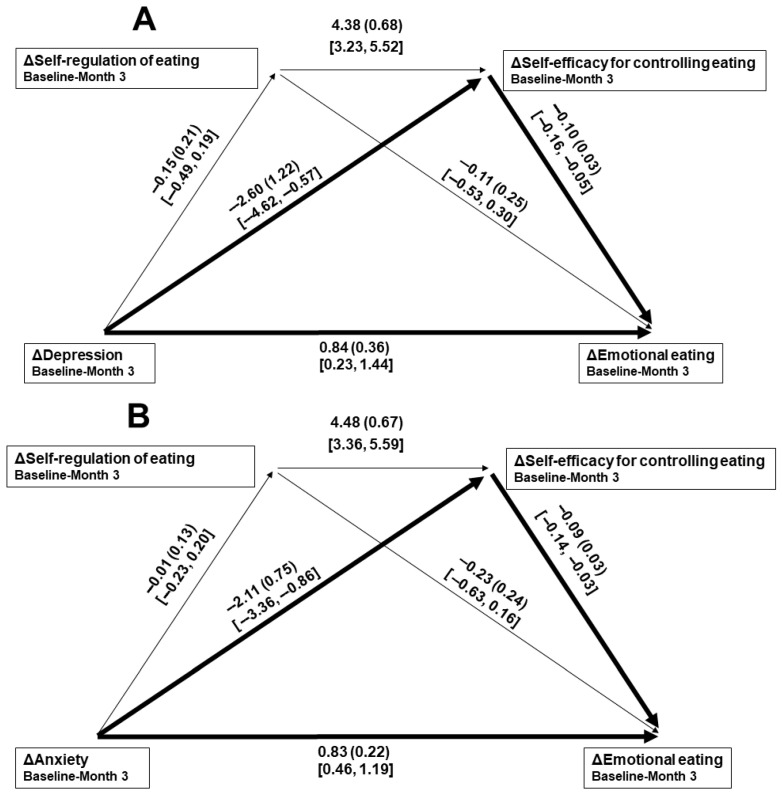
Mediation paths of 3-month changes in depression (**A**) and anxiety (**B**) scores toward emotional eating changes, through changes in self-regulation of eating leading to self-efficacy for controlling eating changes. Δ = change in score. Individual bootstrapped path data are given as B (*SE*_B_) [95% confidence interval]. A heavy line indicates a significant path from mood change toward emotional eating change.

**Figure 2:**
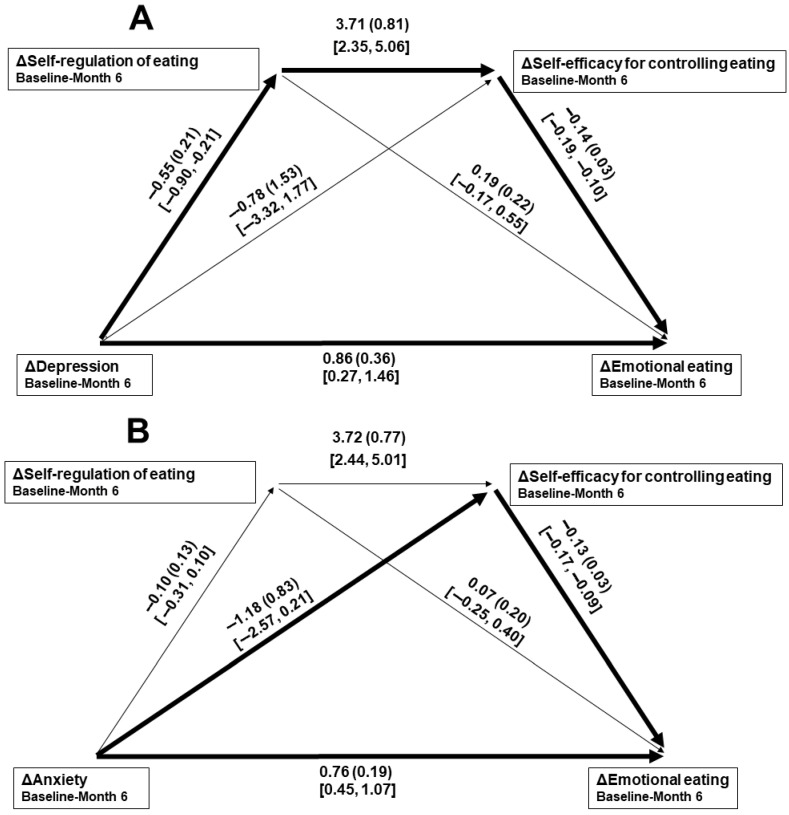
Mediation paths of 6-month changes in depression (**A**) and anxiety (**B**) scores toward emotional eating changes, through changes in self-regulation of eating leading to self-efficacy for controlling eating changes. Δ = change in score. Individual bootstrapped path data are given as B (*SE*_B_) [95% confidence interval]. A heavy line indicates a significant path from mood change toward emotional eating change.

Intercorrelations of changes in the psychosocial variables are given in Table 3. Because five variables were included, a Bonferroni-adjusted significance level of *p* < 0.01 (0.05/5 = 0.01) was set. In the separate mediation paths toward baseline–Month 3 change in emotional eating, rather than the full expected paths, 3-month changes in both depression (B = 0.27, *SE*_B_ = 0.17, 95% CI [0.01, 0.55]) and anxiety (B = 0.18, *SE*_B_ = 0.10, 95% CI [0.04, 0.37]) scores were significantly mediated by change in self-efficacy for controlling eating. Those overall regression models significantly predicted change in emotional eating (*R*^2^s = 0.29 and 0.37, respectively, *ps* < 0.001). Associated path coefficients are given in Figure 1A and Figure 2A. Although only that same significant path was found when considering baseline–Month 6 change in anxiety (B = 0.10, *SE*_B_ = 0.10, 95% CI [0.01, 0.34]), significant mediation of the depression-emotional eating change relationship during that period was only through the hypothesized path of change in self-regulation of eating leading to change in self-efficacy for controlling eating (B = 0.29, *SE*_B_ = 0.12, 95% CI [0.10, 0.51]). Those overall regression models significantly predicted change in emotional eating (*R*^2^s = 0.37 and 0.44, respectively, *ps* < 0.001). Associated path coefficients are given in Figure 1B and Figure 2B. For each mediation path model, the direct relationships between change in the measure of mood (the independent variable) and emotional eating (the dependent variable; Paths *c*′) were significant.

### 3.3. Weight Changes, by Treatment Types

Regarding changes in weight over 3, 6, 12, and 24 months, there were significant treatment group differences at each of those temporal periods, *F* (2, 73)-values = 6.16, *p* = 0.003; 11.39, *p* < 0.001; 8.93, *p* < 0.001; and 3.85, *p* = 0.026, respectively. Follow-up contrasts indicated that the mean weight reductions (*SD*s) associated with the educational treatment of −0.85 kg (2.10), −2.29 kg (2.85), −1.51 kg (4.07), and −1.42 kg (4.27) were less pronounced than mean reductions associated with both the self-regulation treatment of −3.10 kg (3.16), −5.79 kg (3.47), −5.44 kg (4.18), and −6.64 kg (9.50); and the self-regulation + relaxation treatment of −3.26 kg (3.09), −6.04 kg (3.30), −5.75 kg (3.96), and −4.26 kg (5.90) during the same periods of 3, 6, 12, and 24 months. That difference in reduced weight reached statistical significance (*ps* < 0.01), with the exception of the educational and self-regulation + relaxation-based treatment contrast over 24 months (*p* = 0.137). Weight changes in the two self-regulation treatment conditions did not significantly differ during any of the tested temporal intervals (*ps* > 0.20).

### 3.4. Post Hoc Analyses of Psychosocial Predictors of Weight Changes

In post hoc exploratory analyses, multiple regression with simultaneous entry of the independent variables indicated that the interaction of baseline–Month 3 changes in self-regulation and self-efficacy was significantly associated with changes in weight from baseline to Month 3 (*R*^2^ = 0.30), Month 6 (*R*^2^ = 0.15), Month 12 (*R*^2^ = 0.10), and Month 24 (*R*^2^ = 0.07), *ps* ≤ 0.02. The interaction of baseline–Month 6 changes in self-regulation and self-efficacy was also significantly related to changes in weight from baseline to Month 6 (*R*^2^ = 0.12), Month 12 (*R*^2^ = 0.14), and Month 24 (*R*^2^ = 0.18), *ps* < 0.01.

## 4. Discussion

The present results yield important information related to addressing emotional eating via mood changes associated with moderate increases in physical activity. The finding that, as intended, each of the three treatment conditions were not significantly different on their physical activity increases confirmed an important prerequisite for their subsequent contrasts on theory-driven psychosocial and weight variables. In response to Hypothesis 1, it was found that the overall significant improvements in depression, anxiety, emotional eating, and eating-related self-regulation and self-efficacy (large effect sizes) were significantly greater in the two treatments where self-regulation and self-efficacy were emphasized over education. However, the addition of relaxation methods did not significantly increase those effects over either 3 or 6 months. Based on normative data for women [38], this might have been due to a floor effect associated with their identified reductions in depression and anxiety scores.

Consistent with a recent review of 247 studies [20], the results support behavioral (vs. physiological/biochemical) explanations of the relationship between physical activity and mood improvements beyond solely their acute (i.e., just after activity) effects [11]. Based on the present research design—and consistent with social cognitive theory [22,23], self-regulation theory [24], and self-efficacy theory [25]—this implies importance for addressing factors such as nurturing self-regulatory skills and feelings of ability to address personal/environmental challenges within obesity interventions concerned with improving depression, anxiety, and emotional eating. Each of those negative mental states/behavioral propensities have been highly related to obesity [2,58], particularly in women [3].

Hypothesis 2 was partially supported. The expected path from depression and anxiety changes, to change in self-regulation, to self-efficacy change, to emotional eating change was present only when 6-month change in depression was assessed. When 3-month changes in depression and anxiety, and 6-month change in anxiety were evaluated, significant paths were from the mood change, (directly) to self-efficacy change, to emotional eating change. This could be associated with the moderately strong linear association detected between changes in eating-related self-regulation and self-efficacy (βs = 0.50–0.60, *ps* < 0.001; Table 3). The findings extended previous, largely cross-sectional, research [27,28,29,30] through the present use of longitudinal analyses that accounted for changes in theory-based psychological factors within dynamic weight-management processes over both the short- and longer-term. It also informed future treatment process by deconstructing mechanisms of change as had been suggested, but rarely carried out, within the present context [59,60].

Practical implications of the present findings indicate the importance of facilitating increased self-efficacy to enable participants’ control over their emotional eating. Within self-efficacy theory, Bandura [25] proposed four means to improve self-efficacy. The first, and possibly the most crucial, is “mastery experiences.” As described earlier, enabling self-regulation in participants facilitates mastery as newly learned skills empower their self-management amidst lifestyle barriers and challenges, as well as increases in self-appraisals of capability and competence. The present data suggest that physical activity-associated changes in depression and anxiety were more facilitative of self-efficacy changes than self-regulation changes. This supports Bandura’s second theory-based means for self-efficacy increase, namely “psychological states.” Future behavioral obesity interventions should extend those foci to increase self-efficacy for controlled eating via the two additional suggested avenues [25]. The third, “social persuasion” could be advanced through promoting an environment of encouragement and social cohesion via instructors, fellow participants, and peers/family members. Statements suggesting specific areas of goal-related successes should be planned and systematically delivered in an individualized manner by instructors. Finally, “vicarious experience” could be increased by demonstrating to participants success with controlling eating and weight loss in like individuals (i.e., women of a similar age, also initially with obesity). Thus, their confidence in carrying-out challenging behavioral changes could be enhanced.

As in most behavioral weight-loss research, individual participant results varied widely [61]. However, mean weight reductions associated with the two self-regulation treatment groups were generally 2.4 to 3.7 times greater (−5.8% at Month 6; −5.5% at Month 24) than in the educational treatment (−2.4% at Month 6; −1.5% at Month 24). In agreement with previous research reviews [17,56], findings indicated that changes in self-regulation in combination with self-efficacy changes were significant predictors of those weight losses over 3, 6, 12, and 24 months. That finding reinforced their viability as key psychosocial targets within weight-loss treatments.

Although advancements were made within this preliminary field-based research, there were also a number of limitations. For example, the present investigation had: (a) a small sample of volunteer women with obesity who were predominantly White, middle-income, and well-educated; (b) only women who increased their physical activity and, thus, could have been especially motivated; (c) a reliance on self-report measurement, where more objective assessments could be beneficial (e.g., use of accelerometry for physical activity measurement, direct assessment of weight throughout [where self-report was incorporated here at Month 24]); (d) a lack of control of effects from dietary changes; and (e) no true control group to protect from social support, expectation, and other experimental effects. Additionally, data analytic limitations were related to the use of change scores compromising experimental power, aggregating data across groups for regression analyses, and assuming directionality across the tested variables’ relationships. Although the associated hypothesis (Hypothesis 2) was driven by theory and previous research, larger and more diverse samples will enable more thorough explorations of directionalities and moderators in the prediction of emotional eating and weight changes.

Addressing those limitations in future extensions/replications of this small-scale research will both increase confidence in findings and provide evaluation of their stability and generalizability. However, it is recommended that as research seeks to broaden and increase the quality of the present findings, testing should similarly be conducted within field settings so that rapid application of results is made possible. This is especially important given the urgent need for enhanced behavioral approaches to effectively deal with the upwardly trending problem of obesity and its major correlate of emotional eating, as well as the need to understand malleable mechanisms of positive change.

## 5. Conclusions

In summary, findings indicated that, when moderate physical activity is present, improvements in psychosocial factors relevant to emotional eating reduction are more pronounced when obesity treatments focus on the development of self-regulatory skills and self-efficacy than on typical weight-loss educational processes. The present results also suggested that reductions in anxiety and depression are associated with decreased emotional eating through changes in self-efficacy, and the association of changes in self-regulation and self-efficacy. Thus, leveraging physical activity for its psychological-improvement (rather than energy-expenditure) potential was supported, as was a behavioral explanation of the physical activity-mood change relationship. Obesity treatment foci on self-regulation and self-efficacy was also associated with considerably greater amounts of lost weight than commonly applied weight-loss educational methods. As theory- and evidence-driven behavioral methodologies are better developed and administered, it is expected that an improved balance between those and medical interventions will emerge.

## Figures and Tables

**Table 1:** Demographic characteristics of the study sample (N = 76).

Demographic Characteristic	Educational Methods (*n* = 28)	Self-Regulation Methods (*n* = 24)	Self-Regulation + Relaxation (*n* = 24)	F/χ^2^	*p*
Age, *M* (*SD*), years	46.36 (6.25)	44.71 (11.05)	46.63 (10.14)	0.38 ^a^	0.736
Body mass index (BMI), M (SD), kg/m^2^	35.58 (2.89)	35.15 (3.22)	35.26 (3.08)	0.14 ^a^	0.868
Racial/ethnic group, *n* (%)				6.01 ^b^	0.198
White	27 (96.4%)	18 (75.0%)	18 (75.0%)		
Black	1 (3.6%)	3 (12.5%)	3 (12.5%)		
Hispanic	0 (0.0%)	3 (12.5%)	3 (12.5%)		
Yearly family income, *n* (%)				3.27 ^b^	0.774
<US $25,000	4 (14.3%)	1 (4.2%)	2 (8.3%)		
US $25,000–US $49,999	12 (42.9%)	9 (37.5%)	9 (37.5%)		
US $50,000–US $99,999	11 (39.2%)	12 (50.0%)	10 (41.7%)		
≥US $100,000	1 (3.6%)	2 (8.3%)	3 (12.5%)		
Educational level, *n* (%)				1.13 ^b^	0.981
High school/GED	6 (21.4%)	4 (16.7%)	4 (16.7%)		
Some college	6 (21.4%)	5 (20.8%)	5 (20.8%)		
Bachelor’s degree	9 (32.2%)	6 (25.0%)	7 (29.2%)		
More than bachelor’s degree	7 (25.0%)	9 (37.5%)	8 (33.3%)		

^a^ One-way analysis of variance (ANOVA), *F*(2, 73); ^b^ Chi-square test; χ^2^ (4, *N* = 76).

**Table 2:** Changes in study variables and results of mixed-model repeated measures analyses of variance.

Measure	Baseline		Month 3		Month 6		ΔBaseline–Month 3		ΔBaseline–Month 6		Overall Change			Change × Treatment Type		
Treatment Type	*M*	*SD*	*M*	*SD*	*M*	*SD*	*M*	*SD*	*M*	*SD*	*F*(1, 73)	*p*	η^2^_partial_	*F*(1, 73)	*p*	η^2^_partial_
Physical activity																
Educational methods	8.43	8.31	23.32	10.68	25.14	13.87	14.89	5.01	16.71	8.44						
Self-regulation methods	8.85	7.43	26.31	8.07	30.38	13.46	17.46	4.77	21.52	9.88						
Self-regulation + relaxation	8.48	7.65	25.63	8.44	29.69	12.77	17.15	4.79	21.21	8.98						
Aggregated data	8.58	7.73	24.99	9.20	28.23	13.44	16.41	4.94	19.65	9.24	359.84	<0.001	0.83	2.33	0.105	0.06
Depression																
Educational methods	3.14	2.53	1.64	1.39	2.57	1.89	−1.50	2.20	−0.57	1.83						
Self-regulation methods	4.00	3.28	2.04	2.74	1.54	2.02	−1.96	2.73	−2.46	2.19						
Self-regulation + relaxation	4.17	3.47	2.13	2.88	1.53	2.03	−2.04	2.73	−2.63	2.30						
Aggregated data	3.74	3.09	1.92	2.37	1.92	2.01	−1.82	2.52	−1.82	2.28	60.81	<0.001	0.45	7.81	<0.001	0.18
Anxiety																
Educational methods	3.96	2.10	3.54	2.33	3.50	3.12	−0.43	2.39	−0.46	2.73						
Self-regulation methods	4.71	5.00	2.25	2.56	1.88	2.03	−2.46	4.59	−2.83	4.77						
Self-regulation + relaxation	4.63	4.85	2.17	2.33	1.71	1.63	−2.46	4.58	−2.92	4.74						
Aggregated data	4.41	4.07	2.70	2.46	2.42	2.50	−1.71	3.99	−1.99	4.23	19.10	<0.001	0.21	3.03	0.055	0.08
Emotional eating																
Educational methods	27.96	12.46	32.93	9.36	24.46	10.92	4.96	6.70	−3.50	6.39						
Self-regulation methods	27.58	8.93	28.25	12.04	18.58	8.93	0.67	10.03	−9.67	10.03						
Self-regulation + relaxation	27.54	12.28	27.42	9.69	18.42	9.69	−0.13	9.10	−9.13	9.10						
Aggregated data	27.92	12.11	29.50	9.58	20.70	10.23	1.58	8.90	−7.22	8.90	57.27	<0.001	0.44	4.24	0.018	0.10
Self-regulation of eating																
Educational methods	17.71	4.56	21.54	4.94	20.09	4.51	3.83	3.87	2.37	3.98						
Self-regulation methods	17.87	4.04	24.07	3.32	25.03	3.03	6.20	4.65	7.17	3.87						
Self-regulation + relaxation	18.50	4.48	24.48	3.45	25.53	3.28	5.98	4.72	7.03	4.25						
Aggregated data	18.01	4.33	23.27	4.19	23.37	4.45	5.26	4.48	5.36	4.60	141.62	<0.001	0.66	12.14	<0.001	0.25
Self-efficacy for controlling eating																
Educational methods	89.96	32.39	98.18	23.87	96.93	24.35	8.21	29.79	6.96	30.86						
Self-regulation methods	86.50	30.88	115.54	22.91	120.71	22.59	29.04	34.92	34.21	34.90						
Self-regulation + relaxation	89.46	31.14	116.08	24.94	124.10	24.66	26.63	33.56	34.65	33.59						
Aggregated data	88.71	31.14	109.32	25.11	113.02	26.67	20.61	33.62	24.31	35.22	44.24	<0.001	0.38	6.11	0.004	0.14

Educational methods *n* = 28. Self-regulation methods *n* = 24. Self-regulation + relaxation methods *n* = 24. Aggregated data *N* = 76. Δ = score change during the designated period. “Overall change” calculations represent the difference in means change across baseline, Month 3, and Month 6 using data aggregated across all three groups. “Change × treatment type” contrasts those changes, by treatment type (i.e., group). Two-tailed *p*-values are reported. Partial eta-squared (η^2^_partial_) = *SS*_Effect_/(*SS*_Effect_ + *SS*_Error_).

**Table 3:** Intercorrelations of study variables (*N* = 76).

Variable	1	2	3	4	5	6	7	8	9	10
1. ΔDepression B–3	---									
2. ΔDepression B–6	0.67 **	---								
3. ΔAnxiety B–3	0.42 **	0.21	---							
4. ΔAnxiety B–6	0.23 *	0.27 *	0.89 **	---						
5. ΔEmotional eating B–3	0.34 **	0.29 *	0.45 **	0.46 **	---					
6. ΔEmotional eating B–6	0.36 **	0.32 *	0.46 **	0.46 **	0.98 **	---				
7. ΔSelf-regulation of eating B–3	−0.08	0.00	−0.01	0.18	−0.31 *	−0.29 *	---			
8. ΔSelf-regulation of eating B–6	−0.14	−0.29 *	−0.17	−0.10	−0.27 *	−0.25 *	0.68 **	---		
9. ΔSelf-efficacy for controlled eating B–3	−0.24 *	−0.02	−0.26 *	−0.14	−0.49 **	−0.44 **	0.60 **	0.37 **	---	
10. ΔSelf-efficacy for controlled eating B–6	−0.23 *	−0.20 *	−0.20 *	−0.19	−0.61 **	−0.56 **	0.45 **	0.50 **	0.79 **	---

Δ = change in score during the designated period. B–3 = baseline–Month 3. B–6 = baseline–Month 6. * *p* < 0.01. ** *p* < 0.001 (one-tailed tests).

## Data Availability

The data supporting the conclusions of this article will be made available by the first author upon reasonable request.
